# Age-related neuroendocrine, cognitive, and behavioral co-morbidities are promoted by HIV-1 Tat expression in male mice

**DOI:** 10.18632/aging.204166

**Published:** 2022-07-12

**Authors:** Alaa N. Qrareya, Fakhri Mahdi, Marc J. Kaufman, Nicole M. Ashpole, Jason J. Paris

**Affiliations:** 1 Department of BioMolecular Sciences, School of Pharmacy, University of Mississippi, University, MS 38677, USA; 2 Department of Psychiatry, McLean Imaging Center, McLean Hospital/Harvard Medical School, Belmont, MA 02478, USA; 3 Research Institute of Pharmaceutical Sciences, University of Mississippi, University, MS 38677, USA

## Abstract

In the U.S. about half of the HIV-infected individuals are aged 50 and older. In men living with HIV, secondary hypogonadism is common and occurs earlier than in seronegative men, and its prevalence increases with age. While the mechanisms(s) are unknown, the HIV-1 trans-activator of transcription (Tat) protein disrupts neuroendocrine function in mice partly by dysregulating mitochondria and neurosteroidogenesis. We hypothesized that conditional Tat expression in middle-aged male transgenic mice [Tat(+)] would promote age-related comorbidities compared to age-matched controls [Tat(−)]. We expected Tat to alter steroid hormone milieu consistent with behavioral deficits. Middle-aged Tat(+) mice had lower circulating testosterone and progesterone than age-matched controls and greater circulating corticosterone and central allopregnanolone than other groups. Young Tat(+) mice had greater circulating progesterone and estradiol-to-testosterone ratios. Older age or Tat exposure increased anxiety-like behavior (open field; elevated plus-maze), increased cognitive errors (radial arm water maze), and reduced grip strength. Young Tat(+), or middle-aged Tat(−), males had higher mechanical nociceptive thresholds than age-matched counterparts. Steroid levels correlated with behaviors. Thus, Tat may contribute to HIV-accelerated aging.

## INTRODUCTION

In the U.S. ~1.2 million people are living with human immunodeficiency virus type-1 (HIV-1) with men accounting for the majority of cases (~75%; [1]). Although combined antiretroviral therapy (cART) has reduced HIV-associated morbidity and mortality [2–4], patients continue to experience neurological and neuropsychiatric symptoms, collectively called “neuroHIV” [5–8]. This is likely due in part to efflux of some cART regimen from the central nervous system (CNS), high variance in brain accumulation of cART, and non-uniform distribution throughout the brain, all resulting in a lack of linear HIV RNA reduction in the CNS [9–11]. HIV is widely recognized as a disease-driver of aging as age-related disorders present prematurely and/or with greater severity in HIV-infected populations [12–15]. Actions of HIV in the CNS may contribute to premature aging partly by promoting secondary hypogonadism (i.e., hypothalamic and/or pituitary hormonal dysregulation) which is observed in 16% to 25% of young adult or middle-aged HIV-infected men [15–19]. In support, HIV^+^ men transition sooner to andropause [18, 19], characterized by lower total and/or free testosterone (T) seen in patients aged 20–39 years old [16–18, 20], greater circulating sex hormone-binding globulin [16, 17], and greater estradiol (E_2_)-to-T ratios [18] versus healthy age-matched men [21–23]. In the post-cART era, neurotoxic viral proteins persist within the CNS and contribute to mild and moderate forms of neuroHIV [24, 25], yet their contributions to neuroendocrine dysfunction and/or age-related neuroHIV remain unclear and understudied.

One HIV protein secreted from CNS reservoirs (predominantly microglia) that has been well-characterized is the trans-activator of transcription (Tat) [26–28]. Tat is present in the cerebrospinal fluid of cART-treated patients and even when patients achieve complete viral suppression peripherally, reservoirs in the brain continue to secrete Tat [24, 25, 29, 30]. Furthermore, anti-Tat antibodies are associated with reduced cognitive deficits among people living with HIV (PLWH) [31]. These data support the potential role of Tat in the pathophysiology of HIV-associated neurological disorders [24, 25, 29, 30]. Given its persistence in the CNS, Tat may contribute to neuroendocrine dysfunction observed in PLWH, partly due to its well-characterized mitotoxic effects. Tat promotes mitochondrial bioenergetic dysregulation, depolarization, and mitophagy, all of which are known to instigate and exacerbate cellular dysfunction in advanced age [32–37]. Tat-induced mitochondrial dysfunction could also negatively impact neuroendocrine function as mitochondria are the rate-limiting organelle for all steroidogenesis. Indeed, Tat alters lipid bioavailability, limits necessary steroid substrates including cholesterol [38, 39], and promotes the accumulation of ceramides which are inhibitors of steroid-synthesizing enzymes [40]. Using transgenic mice that conditionally express Tat protein, we have observed disruption of the neuroendocrine system, including the hypothalamic-pituitary-adrenal (HPA) and -gonadal (HPG) axes in young adult mice as well as HPG dysregulation in middle-aged female mice [36, 41, 42]. Tat-mediated alterations in steroid hormones may contribute to neurotoxicity. In support, exogenous treatment with T, E_2_, progesterone (P_4_), and 5α-pregnan-3α-ol-20-one (a.k.a., allopregnanolone or alloP) ameliorate Tat-mediated neurotoxicity in cell culture [42–45] and exogenous administration of P_4_ or alloP to Tat-transgenic mice attenuates some neuroHIV-like symptomatology [36, 45, 46]. These steroids, particularly alloP, are found to reduce neurodegeneration in several neurological disease states including Alzheimer’s disease [47, 48]. As such, gonadal hormones may be neuroprotective in middle-aged PLWH. However, the functional effects of Tat on the neuroendocrine milieu of aging males is unknown.

**Figure 1:**
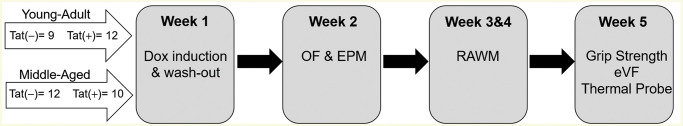
**Time-course of behavioral testing.** In week 1, Tat protein expression was induced in young adult (6–8 mos. old) and middle-aged (11–13 mos. old) male mice via doxycycline administration (30 mg/kg, i.p. for 5 d) followed by two days of doxycycline washout. In week 2, mice were assessed for affective-like behavior in the open field (OF) and elevated plus-maze (EPM). In weeks 3 and 4, spatial memory performance was assessed in a radial arm water maze (RAWM). In week 5, neuromuscular function was assessed by grip strength and nociceptive/analgesic responding was assessed via the electronic-Von Frey and thermal probe tests.

Herein, we investigate the combined effects of aging and HIV-1 Tat expression on the development of neuroHIV-like sequelae in young adult (6–8 months) and middle-aged (11–13 months) male mice to determine whether Tat precipitates age-related dysfunction. We hypothesized that conditional Tat expression in transgenic male mice [Tat(+)] would accelerate the development of (i.e., catalyze earlier presentation) or accentuate (i.e., increase magnitude) age-related affective, cognitive, neuromuscular, and neuropathic pain symptomatology compared to age-matched controls [Tat(–)] (Figure 1). We further expected that Tat would alter the production of circulating and central steroids (T, E_2_, P_4_, corticosterone, or alloP) concurrent with behavioral impairment.

## RESULTS

### Aging and Tat expression altered steroidogenesis in male mice

**Figure 2:**
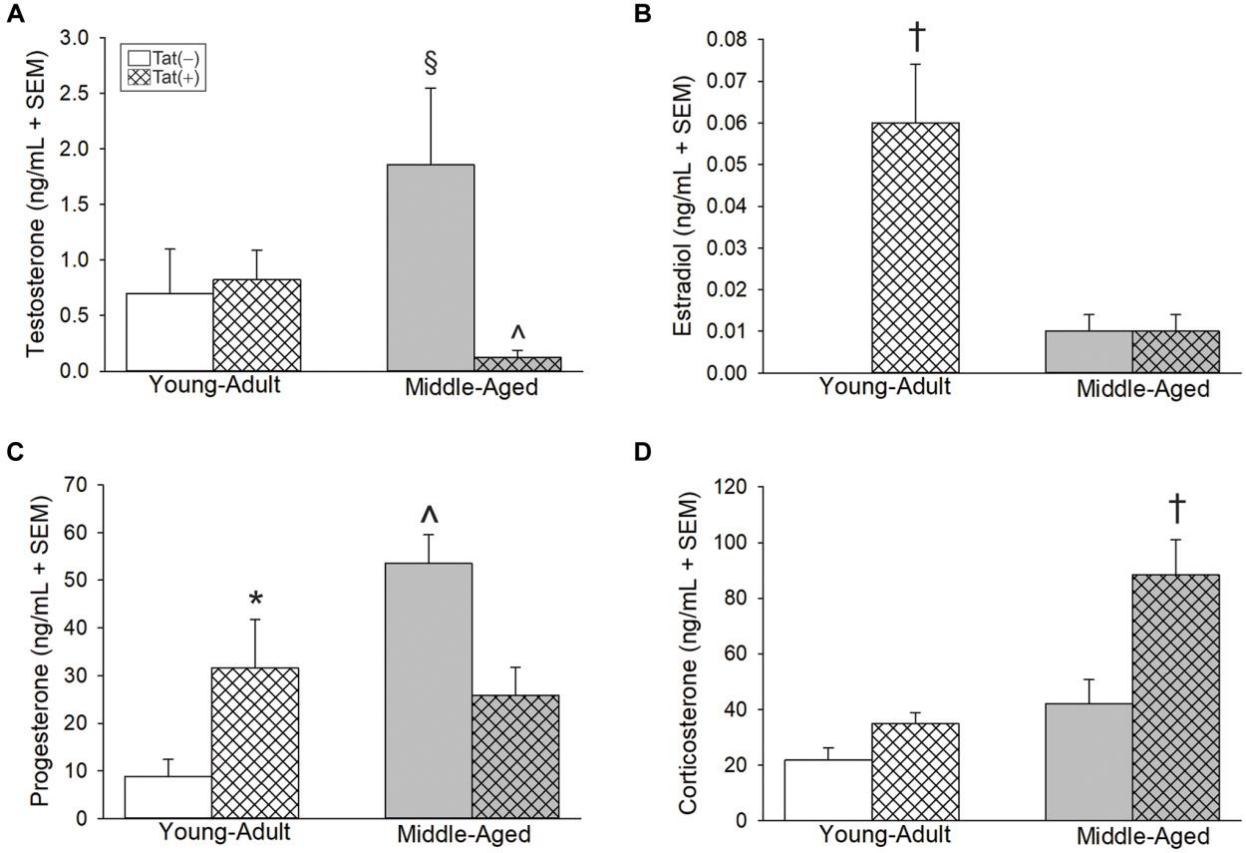
**Circulating steroids (ng/mL) among young adult and middle-aged HIV-1 Tat-transgenic male mice [Tat(+)] or their non-Tat-expressing age-matched counterparts [Tat(−)].** (**A**) Testosterone, (**B**) estradiol, (**C**) progesterone, and (**D**) corticosterone detected in serum. ^§^indicated group differs from young adult Tat(−) controls. ^^^significant interaction wherein indicated group differs from respective Tat(−) controls. ^†^indicated group differs from all other groups. ^*^main effect for Tat(+) mice to differ from Tat(−) controls, (two-way ANOVA, *p* < 0.05).

Aging and Tat expression altered circulating gonadal and adrenal steroid hormones. Older age and Tat expression interacted to influence circulating T [*F*(1,24) = 5.46, *p* < 0.05; Figure 2A]. Middle-aged Tat(−) mice exhibited greater T than did their respective young adult Tat(−) controls (*p* = 0.05; Figure 2A, see §) or their respective middle-aged Tat(+) counterparts (*p* = 0.007; Figure 2A, see ^). Among young adult mice, circulating E_2_ was greater in Tat(+) males compared to all other groups [*F*(1,23) = 34.98, *p* < 0.05; Figure 2B; see †]. Moreover, the calculated E_2_:T ratio (a measure of aromatization) was greatest in young adult Tat(+) mice compared to any other group [*F*(1,20) = 10.39, *p* < 0.05], [E_2_:T ratio: Tat(−)_Young Adult_ = 0 ± 0 ng/mL, Tat(+)_Young Adult_ = 0.06 ± 0.01 ng/mL, Tat(−)_Middle-aged_ = 0.02 ± 0.02 ng/mL, Tat(+)_Middle-aged_ = 0.01 ± 0.01 ng/mL]. E_2_ and the E_2_:T ratio were notably increased with middle-age; however, this did not reach statistical significance (*p* = 0.09, *n.s*.). Circulating P_4_ also interacted with age and Tat expression [*F*(1,27) = 13.16, *p* < 0.05; Figure 2C]. Middle-aged Tat(−) mice demonstrated the greatest circulating P_4_ concentrations of all groups (*p* < 0.0001 – 0.04; Figure 2C, see ^) and young adult Tat(+) mice demonstrated greater P_4_ than their age-matched Tat(−) controls (*p* = 0.02; Figure 2C, see *. Corticosterone was highest among middle-aged Tat(+) mice versus all other groups [*F*(1,23) = 4.69, *p* < 0.05; Figure 2D, see †].

**Figure 3:**
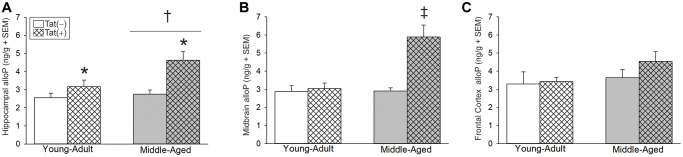
**Central steroid allopregnanolone (ng/g) among young adult and middle-aged HIV-1 Tat-transgenic male mice [Tat(+)] or their non-Tat-expressing age-matched counterparts [Tat(−)].** (**A**) Hippocampal, (**B**) midbrain, and (**C**) frontal cortex allopregnanolone (alloP). ^*^main effect for Tat(−) mice to differ from Tat(−) controls. ^†^main effect for middle-aged mice to differ from young adults. ^‡^indicated group differs from all other groups, (two-way ANOVA, *p* < 0.05).

Older age and Tat expression also interacted to influence brain alloP content. We previously observed that Tat expression increased alloP protein content in the whole brain of young male mice, without changes in serum [36]. However, regional analysis of Tat-provoked alloP in the brain has not been conducted heretofore. In hippocampus, older age [*F*(1,22) = 6.21, *p* < 0.05; Figure 3A, see †] or Tat expression [*F*(1,22) = 14.12, *p* < 0.05; Figure 3A, see *] increased alloP content. In midbrain, age and Tat expression interacted to increase alloP content in middle-aged Tat(+) mice to a greater extent than that seen in all other groups [*F*(1,24) = 13.71, *p* < 0.05, Figure 3B, see ‡]. No significant differences were observed in alloP content within the frontal cortex (Figure 3C).

### Aging or Tat increased anxiety-like behavior among male mice

**Figure 4:**
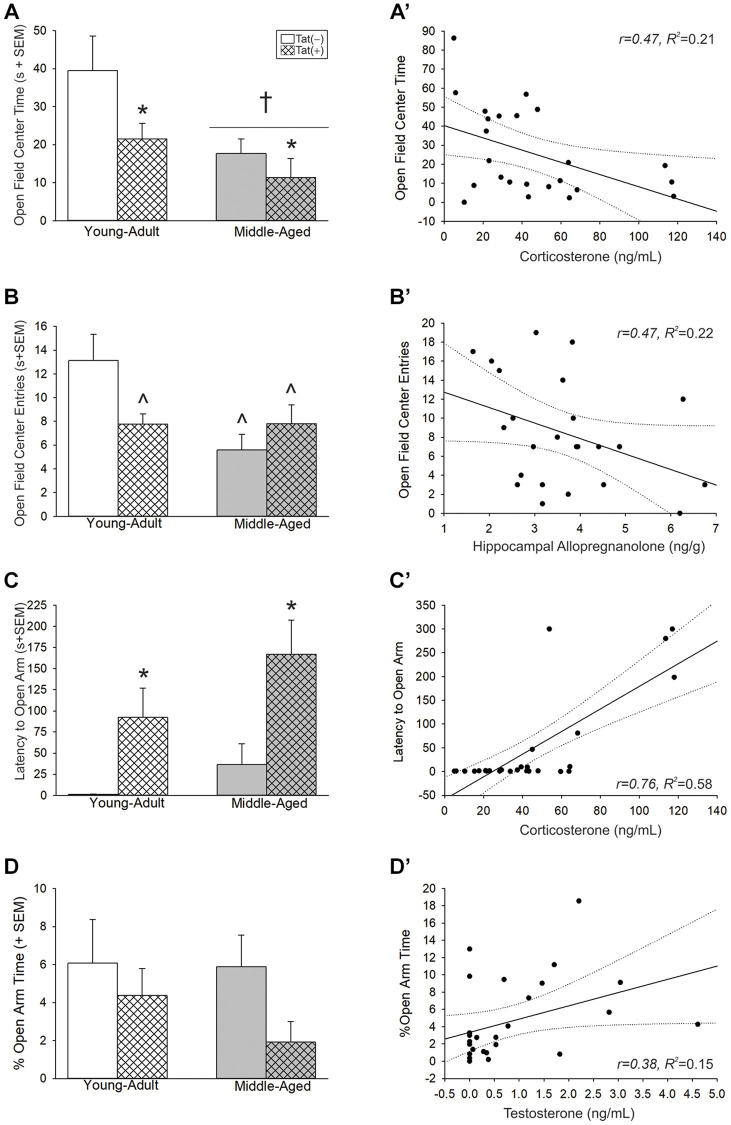
(**A**–**D**) Anxiety-like behavior in the open field and elevated plus-maze and (**A’**–**D’**) simple linear regressions between circulating and central steroid hormones among young adult and middle-aged HIV-1 Tat-transgenic male mice [Tat(+)] or their non-Tat-expressing age-matched counterparts [Tat(−)]. (**A**) Time (s) spent in the brightly-lit center of an open field. (**B**) Numbers of entries made into the center of an open field. (**C**) Latency (s) to enter the open arms of an elevated plus-maze. (**D**) The proportional time spent in the open arms of an elevated plus-maze. Regressions between (**A’**) circulating corticosterone and center time, (**B’**) hippocampal allopregnanolone and center entries, (**C’**) circulating corticosterone and latency to enter open arms, and (**D’**) circulating T and proportional open arm time. ^*^main effect for Tat genotype wherein Tat(+) mice to differ from Tat(−) mice. ^†^main effect for middle-aged mice to differ from young adult mice. ^^^significant interaction wherein indicated group differs from young adult Tat(−) controls. Regression lines (solid) are depicted with 95% confidence intervals (dotted), (two-way ANOVA, *p* < 0.05).

Aging and Tat expression promoted anxiety-like behavior. In the open field, there were main effects for age [*F*(1,35) = 9.14, *p* < 0.05; Figure 4A, see †] and Tat expression [*F*(1,35) = 5.29, *p* < 0.05; Figure 4A, see *], both of which increased anxiety-like behavior and reduced time spent in the center of the open field compared to young adult or Tat(−) counterparts, respectively. As well, there was an interaction wherein age or Tat genotype increased anxiety-like behavior [*F*(1,36) = 5.86, *p* < 0.05; Figure 4B]. Both middle-aged mice and Tat(+) young adult mice made fewer entries into the brightly-lit center of the open field compared to young adult Tat(−) controls (*p* = 0.001–0.027; Figure 4B, see ^). In the elevated plus-maze, we observed a main effect for Tat [*F*(1,38) = 13.13, *p* < 0.05; Figure 4C, see *] to increase the latency to enter the open arms compared to Tat(−) controls. As well, Tat(+) mice demonstrated a notable decrease in the proportion of open arm time compared to Tat(−) mice; albeit, this did not reach statistical significance *(p* = 0.09; Figure 4D). Notably, motor differences were detected for the distance (cm) [*F*(1,36) = 16.82, *p* < 0.05; Table 1] and velocity (cm/s) [*F*(1,36) = 16.85, *p* < 0.05; Table 1] traveled in the open field. Middle-aged or Tat(+) mice demonstrated motor deficits and travelled shorter distances (*p* = 0.0001–0.042), and at lower velocities (*p* < 0.0001–0.041), than did young adult Tat(−) mice. Within the middle-aged group, Tat(−) males travelled shorter distances (*p* = 0.03) and at slower velocities than did Tat(+) mice (*p* = 0.03). No differences were observed in the total arm entries made in the elevated plus-maze (Table 1).

**Table 1:** Motor behavior (open field and elevated plus maze), raw open arm time (elevated plus maze), grip strength, and proportional body weight and peripheral organ wet weights (mean ± SEM) among HIV-1 Tat transgenic [Tat(+)] young adult and middle-aged male mice and age-matched non-Tat-expressing controls [Tat(−)].

	**Young Adult**		**Middle-Aged**	
	**Tat(−) *n* = 9**	**Tat(+) *n* = 9–12**	**Tat(−) *n* = 12**	**Tat(+) *n* = 10**
***Open Field***				
Distance (cm)	1580.40 ± 164.05	1127.45 ± 147.46^^^	907.72 ± 99^^^	1242.34 ± 79.64^^*^
Velocity (cm/s)	5.28 ± 0.55	3.76 ± 0.49^^^	3.03 ± 0.33^^^	4.14 ± 0.27^^^
***Elevated Plus Maze***				
Open Arm Time (s)	13.10 ± 4.96	11.72 ± 3.75	15.34 ± 4.41	3.86 ± 2.10
Total Arm Entries	29.78 ± 3.15	25.58 ± 3.53	25.00 ± 2.66	31.23 ± 4.80
***Grip Strength***				
Forelimbs alone	0.50 ± 0.02	0.35 ± 0.01^^^	0.35 ± 0.02^^^	0.28 ± 0.01^^§^
Forelimbs and hindlimbs	0.62 ± 0.04	0.50 ± 0.01^*^	0.54 ± 0.01^*#^	0.41 ± 0.02^*#^
***Body and Organ Weight***				
Body Weight (g)	31.76 ± 0.96	32.20 ± 1.29	41.92 ± 1.98^‡^	30.50 ± 0.90
Brain weight (%)	1.40 ± 0.06	1.40 ± 0.08	1.14 ± 0.12	1.49 ± 0.10
Heart weight (%)	0.50 ± 0.02	0.71 ± 0.07^^^	0.64 ± 0.07	1.49 ± 0.10^†^
Liver weight (%)	4.48 ± 0.17	4.32 ± 0.33	5.14 ± 0.62	4.92 ± 0.04
Spleen weight (%)	0.28 ± 0.02	0.22 ± 0.04	0.26 ± 0.03	0.62 ± 0.12^‡^

^*^indicates a main effect for Tat(+) mice to differ from Tat(−) mice, irrespective of age. ^#^indicates a main effect for middle-aged mice to differ from young adults, irrespective of genotype. ^indicates a difference from young adult Tat(−) controls. ^†^indicates a difference from young adult Tat(+) mice. ^§^indicates a difference from young adult Tat(+) mice and middle-aged Tat(−) controls.^‡^indicates a difference from all other groups, *p* < 0.05.

Affective-like behavior correlated with steroid fluctuations. In the open field, circulating corticosterone (Figure 4A’; Table 2) and frontal cortex alloP (Table 2) were negatively correlated with the time spent in the center of the open field. Similarly, circulating P_4_ (Table 2) and hippocampal alloP (Figure 4B’; Table 2) were negatively correlated with open field central entries. P_4_ also negatively correlated with the velocity travelled (Table 2). In the elevated plus-maze, circulating corticosterone (Figure 4C’; Table 2) and alloP, within all brain regions examined (i.e., hippocampus, midbrain, frontal cortex; Table 2), positively correlated with the latency to enter the open arms. T levels were also positively correlated with the proportion of time spent on the open arms (Figure 4D’; Table 2).

**Table 2:** Simple linear regressions between circulating or central steroid hormones and behavioral assessments among HIV-1 Tat transgenic [Tat(+)] young adult and middle-aged male mice and age-matched non-Tat-expressing controls [Tat(−)].

**Dependent Variable**	**Independent Variable**	**β**	***t*-value (*df)***	***r***	***R^2^***	***F*-value (*df*)**	***p*-value**
***Open field***							
Center time	Corticosterone	40.34	2.41 (23)	−0.46	0.21	5.82 (1,23)	0.025
	Frontal cortex alloP	57.21	3.29 (23)	−0.57	0.33	10.80 (1,23)	0.003
Center entries	Progesterone	11.31	2.65 (26)	0.47	0.22	7.00 (1,26)	0.01
	Hippocampal alloP	15.64	2.34 (20)	−0.47	0.22	5.50 (1,20)	0.031
	Frontal cortex alloP	13.84	2.06 (22)	−0.41	0.17	4.23 (1,22)	0.05
Velocity	Progesterone	4.54	2.24 (27)	−0.40	0.16	5.01 (1,27)	0.03
Distance	Progesterone	1360.40	2.25 (27)	−0.40	0.16	5.05 (1,27)	0.03
***Elevated plus maze***							
Latency to open arm	Corticosterone	−58.35	5.72 (24)	−0.76	0.58	32.74 (1,25)	<0.0001
	Hippocampal alloP	−126.87	3.89 (24)	0.62	0.39	15.12 (1,25)	0.001
	Midbrain alloP	−63.86	2.87 (26)	0.49	0.24	8.24 (1,27)	0.01
	Frontal cortex alloP	−66.32	2.47 (26)	0.44	0.20	6.12 (1,27)	0.02
% Open arm time	Testosterone	3.35	2.10 (25)	0.38	0.15	4.29 (1,26)	0.05
***RAWM***							
Reference memory error	Estradiol	5.10	2.56 (24)	0.29	0.08	6.53 (1,74)	0.013
Velocity	Progesterone	7.52	4.72 (28)	0.46	0.21	22.23 (1,86)	<0.0001
	Estradiol	8.12	3.74 (24)	0.40	0.16	14.00 (1,74)	0.0004
	Hippocampal alloP	6.30	3.26 (23)	0.35	0.12	10.65 (1,77)	0.002
	Testosterone	8.26	3.10 (25)	0.33	0.11	9.61 (1,80)	0.003
	Corticosterone	8.01	2.47 (24)	0.28	0.08	6.12 (1,74)	0.02
Distance	Testosterone	365.30	2.11 (24)	0.23	0.05	4.44 (1,80)	0.04
***Grip strength***							
% forelimbs alone	Progesterone	1.33	2.04 (26)	−0.37	0.14	4.17 (1,27)	0.05
% Forelimbs and hindlimbs together	Corticosterone	1.82	−2.03 (21)	−0.41	0.17	4.14 (1,22)	0.05
***Nociceptive-like behavior***							
Thermal hyperalgesia	Hippocampal alloP	42.89	2.54 (23)	0.47	0.22	6.45 (1,24)	0.02
***Body and Organs Weight***							
Brain weight (%)	Testosterone	1.43	2.23 (24)	0.41	0.17	5.00 (1,25)	0.04

### Aging or Tat reduced cognitive performance in male mice

**Figure 5:**
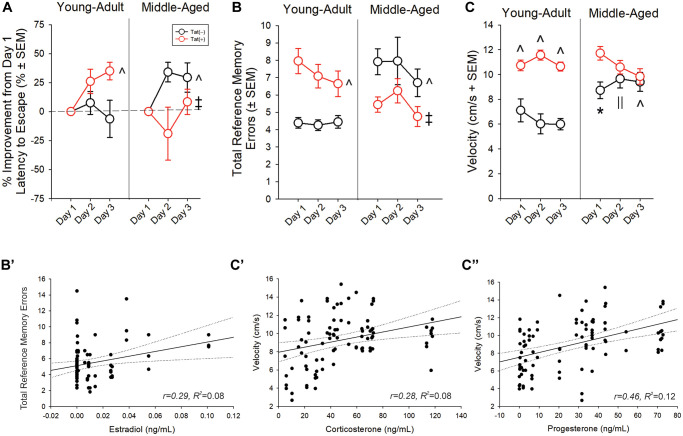
(**A**, **B**) Spatial memory performance and (**C**) swim speed in a radial arm water maze and (**B’**, **C’’**) simple linear regressions for circulating and central steroid hormones among young adult and middle-aged HIV-1 Tat-transgenic male mice [Tat(+)] or their non-Tat-expressing age-matched counterparts [Tat(−)]. (**A**) Proportion of mice that exhibited improvement from day one performance (latency to escape). (**B**) Total frequency of errors. (**C**) Swim speed (cm/s). Simple linear regressions between (**B’**) circulating estradiol and frequency of errors, (**C’**) circulating corticosterone and swim speed, and (**C’’**) progesterone and swim speed. ^*^main effect for Tat(+) mice to differ from Tat(−) mice. ^^^interaction effect wherein indicated group differs from young adult Tat(−) controls. ^||^indicates middle-aged differs from young-adult groups. ^‡^indicated middle-aged Tat(+) groups differs from middle-aged Tat(−) and young adult Tat(+) mice. Regression lines (solid) are depicted with 95% confidence intervals (dotted), (repeated measure ANOVA, *p* < 0.05).

Older age and Tat expression interacted to influence latencies to escape a radial arm water maze [*F*(2,76) = 3.42, *p* < 0.05; Table 3]. Young adult, Tat(−) mice demonstrated longer latencies to escape than young adult Tat(+) mice (day 2; *p* = 0.004) and middle-aged groups (day 3; *p* < 0.001–0.005). Age and Tat expression also interacted to alter the proportional performance improvements from day 1 [*F*(1,35) = 9.46, *p* < 0.05; Figure 5A]. Unexpectedly, young adult Tat(−) mice demonstrated less improvement than did young adult Tat(+) (*p* = 0.022; Figure 5A; see ^) and middle-aged Tat(−) mice (*p* = 0.018; Figure 5A; see ^). Middle-aged Tat(+) mice demonstrated less improvement than did their age-matched, control counterparts (*p* = 0.004; Figure 5A; see ‡) or young adult Tat(+) mice (*p* = 0.006; Figure 5A; see ‡). When assessing the total errors made, age and Tat expression interacted [*F*(1,76) = 17.95, *p* < 0.05; Figure 5B]. Among young adult mice, Tat expression increased the number of errors made (*p* < 0.0001; Figure 5B, see ^). Older age also increased the number of errors made among Tat(−) controls, compared to their young adult counterparts (*p* < 0.0001; Figure 5B, see ^). Contrary to expectation, middle-aged Tat(+) mice made fewer errors than did their respective Tat(−) controls or their young adult Tat(+) counterparts (*p* = 0.001–0.006; Figure 5B; see ‡). This may have been related to differences in swim speed. Among the middle-aged group, Tat(+) mice swam faster than their age-matched, or young-adult, Tat(−) counterparts only on day 1 (*p* < 0.0001–0.002; Figure 5C, see *). Middle-aged mice were faster than their young adult controls on days 2 (*p* < 0.0001–0.03; Figure 5C, see ||) and 3 (*p* < 0.0001; Figure 5C, see ^). Fewer errors made by middle-aged Tat(+) mice may be a consequence of increased anxiety and faster latencies to escape (Table 3). In support, despite observing no difference in the distance travelled (Table 3), a significant interaction between age, Tat expression, and day of testing was observed for swim speed [*F*(2,78) = 5.82, *p < 0.05*; Figure 5C]. Among young adults, Tat(+) mice swam faster than their Tat(−) counterparts over all three days (*p* < 0.0001–0.0003; Figure 5C, see ^).

**Table 3:** Latency to escape (s) and distance travelled (cm) in a radial arm water maze among HIV-1 Tat transgenic [Tat(+)] young adult and middle-aged male mice and age-matched non-Tat-expressing controls [Tat(−)].

**Latency to Escape (s)**	**Young adult**		**Middle-aged**	
	**Tat(−) *n* = 8–9**	**Tat(+) *n* = 12**	**Tat(−) *n* = 12**	**Tat(+) *n* = 10**
Day 1	47 ± 6	36 ± 3	49 ± 6	37 ± 4
Day 2	49 ± 8	27 ± 2^*^	35 ± 6	37 ± 4
Day 3	50 ± 5	25 ± 3^^^	31 ± 6^^^	33 ± 4^^^
Distance (cm)				
Day 1	372 ± 31	386 ± 29	410 ± 30	387 ± 30
Day 2	318 ± 32	320 ± 27	334 ± 37	380 ± 31
Day 3	349 ± 48	271 ± 23	307 ± 29	298 ± 31

^*^indicates an effect for young adult Tat(+) mice to differ from young adult Tat(−) mice. ^^^indicates a difference from young adult Tat(−) controls. *p* < 0.05.

Radial arm water maze performance correlated with circulating and central steroid levels. Circulating E_2_ was positively correlated with the total reference memory errors (Figure 5B’; Table 2). Corticosterone (Figure 5C’; Table 2), P_4_ (Figure 5C’’; Table 2), E_2_ (Table 2), hippocampal alloP (Table 2), and T (Table 2) were positively correlated with swim speed. T was negatively correlated with the total distance travelled (Table 2).

### Aging and Tat reduced neuromuscular strength among male mice

**Figure 6:**
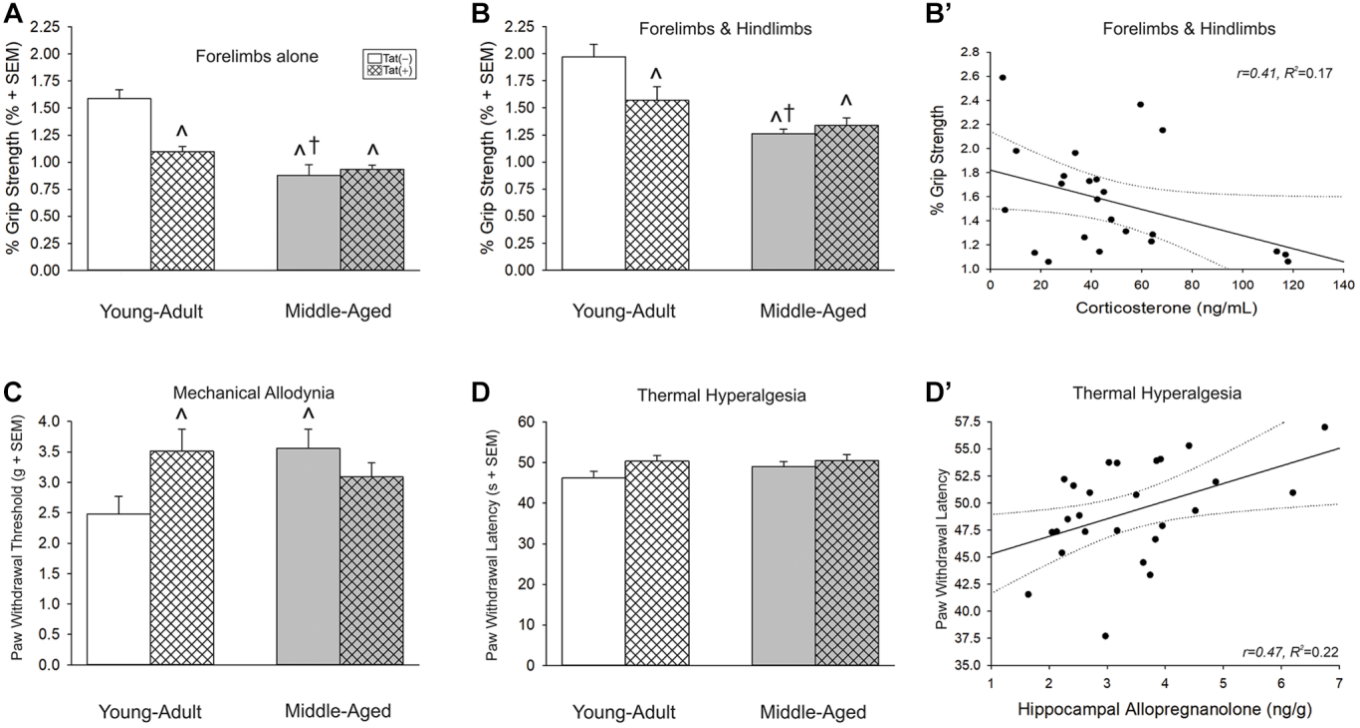
(**A**, **B**) Grip strength, (**C**) mechanical allodynia, and (**D**) thermal hyperalgesia and (**B’** and **D’**) simple linear regression for circulating and central steroid hormones among young adult and middle-aged HIV-1 Tat-transgenic male mice [Tat(+)] or their non-Tat-expressing age-matched counterparts [Tat(−)]. Grip strength threshold for (**A**) forelimbs alone or (**B**) forelimbs and hindlimbs together. (**C**) Paw-withdrawal threshold (*g*) in an electronic Von Frey test. (**D**) Paw-withdrawal latency (s) in a thermal probe test. Simple linear regressions between (**B’**) circulating corticosterone and forelimbs and hindlimbs together or (**D’**) hippocampal allopregnanolone and paw-withdrawal latency. ^*^main effect for Tat(+) mice to differ from Tat(−) mice. ^†^main effect for middle-aged mice to differ from young adults. ^^^significant interaction wherein indicated group differs from young adult Tat(−) controls. Regression lines (solid) are depicted with 95% confidence intervals (dotted), (two-way ANOVA, *p* ≤ 0.05).

Advanced age and Tat interacted to reduce grip strength when assessed either by forelimbs alone (normalized to body weight) [*F*(1,37) = 13.68, *p* < 0.05; Figure 6A] or by forelimbs and hindlimbs together (normalized to body weight) [*F*(1,36) = 7.00, *p* < 0.05; Figure 6B]. In either measure, middle-aged or Tat(+) mice were weaker than young Tat(−) controls (*p* < 0.0001–0.004, Figure 6A, 6B, see ^). As well, middle-aged Tat(−) mice were weaker than young adult Tat(+) mice (*p* = 0.02–0.03, Figure 6A, 6B, see †). Grip strength assessment without body weight normalization yielded comparable effects (Table 1). There was an interaction between age and Tat exposure when assessing forelimbs alone [*F*(1,37) = 9.24, *p* < 0.05; Table 1] such that all groups were weaker than young adult Tat(−) controls (*p* < 0.0001). As well, there was a “stair-step” effect wherein middle-aged Tat(+) mice were weaker than their own middle-aged Tat(−) controls (*p* = 0.002) or young adult Tat(+) mice (*p* = 0.002). There were also main effects for age [*F*(1,37) = 12.14, *p* < 0.05; Table 1] or Tat exposure [*F*(1,37) = 25.64, *p* < 0.05; Table 1] to reduce combined forelimb and hindlimb grip strength.

Grip strength was also correlated with circulating steroids. Corticosterone was negatively correlated with neuromuscular function as assessed by the forelimbs and hindlimbs together (Figure 6B’; Table 2) and P_4_ negatively correlated with neuromuscular function as assessed by the forelimbs alone (Table 2).

### Aging and Tat altered nociceptive tolerance in male mice

Older age and Tat expression interacted to influence the threshold for paw withdrawal in response to a mechanical stimulus [*F*(1,37) = 5.87, *p* < 0.05; Figure 6C]. Unexpectedly, middle-aged Tat(−) and young adult Tat(+) mice exhibited a greater mechanical threshold than did young adult Tat(−) controls (*p* = 0.02–0.03; Figure 6C, see ^). No differences were observed in thermal hyperalgesia among middle-aged or Tat-exposed mice (Figure 6D); however, hippocampal alloP was positively correlated with the latency to paw withdrawal from a thermal stimulus (Figure 6D’; Table 2).

### Aging and Tat influenced body and peripheral organ weight

Aging and Tat expression interacted to influence body weight [*F*(1,37) = 17.00, *p* < 0.05; Table 1] such that middle-aged Tat(−) mice were heavier than all other groups (*p* = 0.0001–0.004). There was also an interaction effect between age and Tat to increase proportional heart wet weights [*F*(1,25) = 8.41, *p* < 0.05; Table 1] wherein young adult Tat(+) mice had greater proportional mass than their age-matched Tat(−) counterparts (*p* = 0.011) or middle-aged Tat(+) mice (*p* = 0.04). The proportional spleen wet weight [*F*(1,24) = 12.33, *p* < 0.05; Table 1] was greatest in middle-aged Tat(+) mice compared to all other groups (*p* = 0.0001–0.0003). We did not observe differences in the proportional weights of brain or liver (Table 1). However, circulating T was positively correlated with proportional brain wet weight (Table 2).

## DISCUSSION

HIV-infected individuals contend with an accelerated onset of age-related diseases and disorders [49–51]; however, the pathophysiology underlying accelerated aging is poorly understood. Findings from this study support the hypothesis that aging and HIV-1 Tat protein exert independent and interactive effects to induce neuroendocrine dysfunction which coincide with neuroHIV-like symptomatology in male mice. Consistent with observations in HIV^+^ patients [16–17, 20], Tat expression in mice reduced circulating T among middle-aged males. Moreover, HIV^+^ men (ages 18–70 y/o) experience increased E_2_: T ratios [18], and we find that Tat expression recapitulates this endophenotype in male mice. Tat induction also elevated circulating P_4_ in young adult mice, an endocrine profile observed in our middle-aged controls suggesting that Tat expression may accelerate the onset of endocrine dysregulation. These preclinical data support the notion that exposure to Tat is sufficient to induce changes in the HPG axis that are consistent with clinical phenotypes. We observed further changes in the HPA axis as the animals aged. Tat expression elevated circulating corticosterone and central alloP in the hippocampus and midbrain of middle-aged mice. These neuroendocrine responses correlated with Tat-induced behavioral deficits, confirming our prior findings of Tat-disrupted alloP neurosteroidogenesis [36] and HPA axis dysregulation [52] and revealing their long-term consequences in the development of age-related neurological comorbidities. In addition, Tat altered peripheral organs, inducing an increase in the proportion of heart wet-weight. In particular, middle-aged Tat(+) mice had the greatest heart wet-weight of any group. These data are consistent with premature heart remodeling to promote hypertrophy; heart disease is notably a leading cause of mortality among PLWH [53] and its incidence increases with age [54, 55]. Middle-aged Tat(+) mice also had greater spleen weights than other groups which could be associated with several age-related comorbidities including lymphoma. Future pathological studies may expand on these findings.

Mood and cognitive disorders are common among HIV^+^ patients [49, 56–59] and their prevalence increases with age [60] and with endocrine dysfunction [61, 62]. Irrespective of HIV status, age-related androgen decline is associated with a greater risk of developing dementia, Alzheimer’s disease [63, 64], and cognitive impairment in men [65]. In the pre-cART era, hypogonadal men had higher depression scores than eugonadal men [61]. Among individuals having progressed to AIDS, risk for depression was greater than in eugonadal HIV^+^ men [66]. While, endocrine disorders have declined in the post-cART era, secondary hypogonadism is still prevalent among HIV^+^ patients (16–25%) [18, 21, 67, 68]. Testosterone insufficiency is associated with depression/apathy, cognitive impairment, sexual dysfunction, fatigue, reduced muscle strength, and increased risk for HIV-associated lipodystrophy [19, 20]. Herein, we find that older age and/or Tat expression increase anxiety-like behavior in an open field and in an elevated plus-maze. In both tasks, Tat expression engendered an anxiety-like phenotype in young adults comparable to that observed in middle-aged mice, consistent with accelerated aging. Testosterone positively correlated with anti-anxiety-like behavior on the elevated plus maze supporting a role for androgens to improve mood. On the RAWM, Tat expression increased the frequency of errors made throughout the learning phase of the task, consistent with prior demonstrations of Tat-impaired spatial memory [41, 69–74]. These data extend prior findings to demonstrate that older age also increases errors, an effect that was positively correlated with greater circulating E_2_. The distance travelled to escape the RAWM was negatively correlated with circulating T, further supporting the benefits of a eugonadal state. Contrary to expectation, Tat-exposed middle-aged mice made fewer errors than their age-matched Tat(−) counterparts. This may reflect greater activation of the HPA axis in older Tat(+) mice. In support, middle-aged Tat(+) mice were faster than controls on day 1 of the RAWM and had greater corticosterone levels that also positively correlated with swim speed. This group also had the lowest circulating T and lower body weight than their non-Tat expressing middle-aged counterparts supportive of a reduced anabolic state. Consistent with the possibility of a generalized HPG/HPA activation in response to stress, circulating steroids and hippocampal alloP levels were positively correlated with RAWM swim speed. Thus, age and Tat effects on HPG/HPA axis function influenced ‘performative measures’ (i.e., open field and swim velocity) and these were associated with affective and cognitive dependent measures.

In the present work, an attenuated HPA axis was associated with improved neuromuscular function and hyperalgesia. Moreover, young adult Tat(+) mice demonstrated grip strength that was commensurate to that of middle-aged Tat(−) or Tat(+) mice suggestive of Tat’s capacity to accelerate age-related neuromuscular dysfunction. These observations correlated negatively with circulating corticosterone and P_4_. In HIV^+^ patients, neuromuscular deficits occur concurrent with disease progression and the onset of painful neuropathies [75]. As such, we also assessed mechanical antinociception and thermal hyperalgesia. We observed that aging or Tat expression increased pain-like thresholds among mice. While, this is contrary to the expectation of peripheral neuropathy, we previously observed similar effects in young adult male Tat(+) mice, which had greater antinociceptive thresholds compared to their Tat(−) counterparts [76]. This effect may involve peripheral sensory damage given evidence for intra-epidermal nerve fiber regression [77], which may impact sensation. In support, ~50% of HIV^+^ patients experience neuropathic pain and/or paresthesias, the latter of which are difficult to assess in mouse models [78]. We did not observe any thermal hyperalgesia, consistent with prior reports [41, 77], but hippocampal alloP was positively correlated with greater thermal thresholds. Several factors may contribute to neuropathic pain in the HIV^+^ population, including neurotoxic antiretroviral treatment (nucleoside reverse transcriptase inhibitors; albeit, used more sparingly in developed nations [79]), or metabolic syndrome [80], and age [81, 82]. As well, HIV-1 proteins beyond Tat, including the envelope glycoprotein 120 (gp120), likely contribute to neuropathic pain. For example, in cultured dorsal root ganglion neurons, gp120 exerts chemokine-like effects via increased neural excitation and substance P release [83]. Intradermal injection of gp120 induces allodynia [83] and reduces epidermal nerve fiber density [84]. Discrepancies in findings for Tat-induced mechanical allodynia may also involve sex differences. We previously found that middle-aged Tat(+) females have reduced mechanical pain thresholds compared to controls [41], in contrast to current findings in middle-aged Tat(+) males. Bagdas et al. (2020) observed similar effects in a cohort of age-matched young adult Tat-transgenic mice.

Beyond typical hormone replacement therapies, neurosteroid-based therapeutics may hold promise for numerous age-related diseases and disorders. In rodents, androgen replacement therapy including testosterone and its neuroactive metabolite, 5α-androstan-3α, 17β-diol (a.k.a. 3α−androstanediol), reduces anxiety- and depression-like behavior and improves neurocognitive performance [85–87]. Similarly, exogenous alloP improved hippocampal-dependent learning and memory among aged male mice including the triple-transgenic Alzheimer’s model [87, 88]. We have found that exogenous alloP attenuated affective dysfunction, mitotoxicity, and neurotoxicity associated with HIV-1 Tat [36, 45]. Together, these data support the notion that maintaining the endocrine milieu of the CNS promotes resilience to neurodegenerative insults, including those associated with older age. In support, we recently found that T declines with age in the medial prefrontal cortex and striatum of Tat-expressing transgenic mice, concurrent with decrements in excitatory and inhibitory neurotransmitters as well as endogenous antioxidants detected with magnetic resonance spectroscopy [89]. These findings are consistent with clinical observations of cognitive impairment [90] that occurred in parallel with cerebral metabolic disturbances including a reduction of N-acetyl aspartate and an increase of choline and myoinositol, markers of inflammation, in HIV^+^ patients [29, 90, 91]. Further, older age and HIV tended to exert additive effects at reducing subcortical gray matter in regions that included amygdala, caudate, and corpus callosum in one study [92] and altered β-amyloid deposition in another [93]. These findings are consistent with observations of accumulated hyperphosphorylated Tau in the hippocampus of HIV^+^ patients [94]. Others have found synergistic effects of older age and HIV on the exacerbation of verbal memory deficits [95]. Given the potential protective benefits of neurosteroids for neurodegenerative disease states ranging from Alzheimer’s [96] to neuroHIV, neurosteroids may serve as scaffolds for a new generation of age-based therapeutics.

The present study had some limitations. We measured total circulating steroid content but did not assess the free, bioavailable fraction. Given increases in sex hormone-binding globulin among HIV^+^ patients [16, 17, 67, 97, 98], future studies will discern the amount of bioavailable androgen. As well, we have recently found that middle-aged female mice also display HPG dysregulation that correlated with some behavioral deficits [41]. Together, these data support the assessment of exogenous steroid hormone treatment for potential benefits using aged models. While the present study did not include mice greater than 13 months of age, the current work highlights Tat’s capacity to precipitate several characteristic phenotypes of advanced age on its own; thus, suggesting that Tat accumulation can decrease health span within mice.

The current work provides potentially actionable information that could help to limit premature or accentuated aging in the context of HIV. The hypogonadism observed in clinical populations may reflect substantial neuroendocrine dysfunction that can be readily assessed in preclinical models. In support, the formation of neurosteroids are dysregulated in post-mortem HIV^+^ brains [99] and in the circulation of HIV^+^ patients [100]. The latter included the dehydroepiandrosterone sulfate-to-cortisol ratio, a biomarker for HPA dysregulation, which was associated with depressive symptomatology [100]. As well, T replacement improves mood disorders and lean body weight in HIV-infected men [66, 101, 102]. In conclusion, our data suggest that older age and Tat expression exert independent and interactive effects to worsen neuroendocrine, affective, cognitive, and neuromuscular comorbidities. Novel steroid replacement therapies may be useful adjunctive therapeutics to cART in the aging HIV^+^ population.

## MATERIALS AND METHODS

All experimental procedures were approved by the Institutional Animal Care and Use Committee at the University of Mississippi and conduced in accordance with the National Institutes of Health Guide for Care and Use of Laboratory Animals (NIH Publication No. 85–23) ethical guidelines.

### Animals and housing

Young adult (6–8 months old; n_Tat(−)_ = 9, n_Tat(+)_ = 12) or middle-aged (11–13 months old; n_Tat(−)_ = 12, n_Tat(+)_ = 10) HIV-1 Tat-transgenic male mice were generated in the vivarium at the University of Mississippi (University, MS). These mice were founded on a C3H × C57BL/6J background and have been repeatedly back-crossed to C57BL/6J [103, 104]. In Tat(+) mice, the Tat_1-86_ protein is conditionally-expressed via administration of doxycycline, as described [103]. Tat(−) mice express the rtTA transcription factor necessary for *tat* expression, but lack the *tat* transgene [103]. Mice were group-housed (3–4 per cage) in a temperature- and humidity-controlled facility on a reversed 12:12-h light:dark cycle (lights off at 09:00 h). Food and water were available *ad libitum.* Animal housing rooms were specific pathogen free (parvovirus, helicobacter, etc.). Mouse ages were selected from prior work that associates human age with murine development, finding one year of human life equivalent to nine days in the moues during adulthood [105]. These ages are consistent with prior work comparing young adult and middle-aged mice [106–108].

### HIV-1 Tat induction

To conditionally express the HIV-1 *tat* transgene (or not), Tat(+) mice and their Tat(−) counterparts received doxycycline (Dox) hyclate (30 mg/kg; Cayman Chemical, Ann Arbor, MI) intraperitoneally (i.p.) once daily for five consecutive days, followed by a 2-day wash-out period to rule out non-specific effects of Dox (Dox *t*_1/2z_ = 5–6 h; [109]).

### Behavioral assessment

Mice were tested over the course of four-weeks using several behavioral assessments in the following order: open field, elevated plus maze, radial arm water maze, grip strength, electronic Von Frey (eVF), and thermal probe. A 48-h break occurred between each behavioral test except for neuropathic-like pain assessments, eVF and thermal probe, which were conducted on the same day (Figure 1). Mice were handled daily before behavioral assessments, which were conducted ~1 h into their dark phase. Thirty min prior to testing, mice were transferred to a testing room and habituated to 70dB of white noise. Behavioral apparatus was cleaned with 70% ethanol between trials to avoid olfactory bias. All behavioral assessments were tracked and digitally encoded by EthoVision animal tracking software (Noldus, Leesburg, VA, USA).

### Open field

The open field test was used to assess anxiety-like behavior and ataxia in rodents [46, 110]. Mice were placed in the center of the brightly-lit area of the open field apparatus (40 × 40 × 35 cm) and allowed to freely explore the apparatus for 5 min. Greater time spent, and more entries made into, the brightly-lit center area were used as indices of anti-anxiety-like behavior. Total distance moved (cm) and velocity (cm/s) were considered indices of locomotor activity [46, 110].

### Elevated plus maze

The elevated plus maze was used to assess anxiety-like behavior [41, 111]. Briefly, the maze consisted of two cross-arms (two open and two enclosed arms; 61 × 5 cm ea.) that were elevated from the floor (37.5 cm). Mice were placed in the plus-maze center area facing the open arm and allowed to freely explore for 5 min. A shorter latency to enter the open arm and a greater proportional time spent on the open arms were considered indices of greater anti-anxiety-like behavior. The total number of entries made into arms was used as an index of locomotor activity [41].

### Radial arm water maze

The radial arm water maze (RAWM) was used to assess learning and spatial memory [112, 113]. The maze consisted of eight arms (24 × 8 cm) attached to a central area (18 cm). Briefly, the maze was filled with room-temperature tap water (equilibrated overnight) and a hidden platform (6 × 8 cm) was located at the end of a predetermined arm (goal arm). Mice completed 6 trials daily for 3 days. In each trial, mice were placed in a randomized, predetermined (starting arm) and allowed to swim freely for 90 s to reach a hidden platform. When mice failed to locate the hidden platform, they were gently guided by the investigator to the platform and allowed to remain for 15 s. The starting arm was randomized for each trial. A shorter time to locate the platform and fewer total errors made were considered indices of greater spatial cognitive performance [112, 113]. To assess the proportional improvement from day 1, the following calculation was performed: 1 – (latency to escape on days 2 or 3/latency to escape on day 1) × 100 [41]. Entries into an incorrect arm were counted as errors. Total distance (cm) and velocity (cm/s) were used as indices of locomotor activity.

### Grip strength

Grip Strength was used to assess neuromuscular function in rodents as previously described [41, 114]. Briefly, mice were suspended by the tail and allowed to grab a metal grid bar connected to a transducer that measured peak force (*g*). Mice were gently pulled by the base of the tail horizontally away from the bar until grip was lost. The strength of forelimbs, alone or in combination with hindlimbs, was measured. Each mouse was tested in five trials with a 1 min break between trials to prevent fatigue. The mean of five trials was used as the grip strength score. Grip strength values were normalized to body weight and considered as an index of neuromuscular function [41, 114].

### Electronic Von Frey

The electronic Von Frey (eVF; Top Cat Metrology) test was used to measure mechanical allodynia as previously described [41, 115]. Mice were allowed to habituate for 15 min on an elevated wire mesh. A series of eVF probes was applied to the hind paw (at the middle plantar surface) with force gradually increasing until paw withdrawal occurred. Each mouse was assessed in eight trials alternating between right and left paws (4 for each side) with a 3-min inter-trial interval. The mean of 4 trials for each paw was calculated. A lower mechanical threshold indicated an increase in the allodynic response [41, 115, 116].

### Thermal probe test

A thermal probe test was used to evaluate thermal hyperalgesia in rodents as previously described [41, 117]. A radiant heat stimulus was applied to the mid-plantar surface of the hind paw. The heat source was increased from room temperature to 60°C at a rate of 2.5°C/s. The test was stopped once a paw withdrawal occurred. Each mouse was assessed in 4 trials (2 for the right and 2 for the left paw) by alternating between right and left paws. Mice had 3–5 min breaks between trials. Paw withdrawal latencies were averaged and used as an index of hyperalgesia [41, 117].

### Central and circulating steroid assay

#### Tissue collection

Two days following behavioral assessments, a subset of animals (young adult; n_Tat(−)_ = 9, n_Tat(+)_ = 8) or middle-aged; n_Tat(−)_ = 6, n_Tat(+)_ = 8) was sacrificed via cervical dislocation followed by rapid decapitation. Brain, heart, liver, and spleen were collected, weighed and stored at −80°C for later analysis. Blood was collected and centrifuged at 13,500 × *g* (4°C) for 20 min. Serum and all tissues were stored at −80°C. Remaining mice underwent perfusion for imaging to be reported elsewhere.

### Enzyme-linked immunosorbent assay (ELISA)

Steroid extraction from serum was achieved via diethyl ether snap-freezing as previously described [42, 45]. Briefly, serum was transferred to glass borosilicate culture tubes and the total volume was measured. One mL of ice-cold anhydrous diethyl ether was added to all samples which then underwent snap-freeze for 30 s. Supernatant was collected and allowed to evaporate to dryness overnight in a fume hood. Using ELISA extraction buffer (Neogen Life Sciences, Lexington, KY, USA), samples were reconstituted to 5× their original volume. Circulating steroid hormones (E_2_, P_4_, T, and corticosterone) were analyzed using ELISA kits according to manufacture instructions (Neogen Life Sciences, Lexington, KY, USA) and previous methods [42, 45]. Steroids were reconstituted with the extraction buffer at 1:5, 1:25, 1:100, and 1:50 dilutions for E_2_, P_4_, T, and corticosterone, respectively. Hydrolysis of substrate was measured at 560 nm (optic density) using a CLARIOstar microplate reader (BMG Labtech Inc., Cary, NC, USA). Steroid hormone concentrations were calculated using a curve-fitting routine (4-parameter). The antibodies supplied reported a 100% cross-reactivity with the steroids of interest. The E_2_ antibody exerts low cross-reactivities (<1%) with other steroids. The P_4_ antibody has 2.5% and <2% cross-reactivities with deoxycorticosterone and other steroids, respectively. The T antibody exhibits 100% cross-reactivity with dihydrotestosterone, 16.12% cross-reactivity with T-glucuronide, and <1% cross-reactivity with other steroids. The corticosterone antibody exhibits cross-reactivities with deoxycorticosterone (38%), 6-hydrocortisone (19%), P_4_ (5.1%), and tetrahydrocortisone (2.7%), as reported by the manufacture (Neogen Life Sciences, Lexington, KY, USA). Standards ranged from (0.02–2 ng/ml), (0.4–10 ng/ml), (0.002–0.2 ng/ml), and (0.04–10 ng/ml) for E_2_, P_4_, T, and corticosterone, respectively.

### Ultra performance liquid chromatography (UPLC) - mass spectrometry (MS)

Brain regions (hippocampus, midbrain, and frontal cortex) were grossly-dissected, wet weighed, and stored at −80°C until UPLC-MS/MS was conducted. Charcoal-stripped brain tissues derived from Tat(−) and Tat(+) mice were used for calibration and quality control. Samples were homogenized in 100 μl of PBS (pH 7.4) and steroid extraction was achieved via protein precipitation. Samples were precipitated with 100 μl of acetonitrile followed by vortexing for two min and centrifugation for ten min (14,000 rpm). Following centrifugation, supernatants were mixed with 50 μl of derivatizing solution (20 mg/mL 2-hydrazinopyridine prepared in 0.5% trifluoroacetic acid ethanol solution) and incubated for 1 h at 60°C. 20 μl of the internal standard solution (LLOQ: 0.25 ng/ml) was added and vortexed. For analysis, 2 μl of sample was injected onto the UPLC-MS/MS instrument [118].

### Statistical analyses

Behavioral endpoints (open field, elevated plus-maze, grip strength, eVF and thermal probe) were analyzed via two-way analyses of variance (ANOVA) using age-group and Tat genotype as factors. Data obtained from radial arm water maze were analyzed via repeated measures ANOVA with testing day as the within-subjects factor and age-group and Tat genotype as the between-subjects factors. Main effects were delineated using Fisher’s Protected Least Significant Difference *post hoc* tests to determine group differences. Interactions were delineated via the assessment of simple main effects and main effect contrasts with alpha (0.05) controlled for family-wise error. Outliers were determined by Dixon’s test and were excluded. All data were considered significant when *p* ≤ 0.05.

### Data and materials availability

Data are available upon request.
